# Psychische Langzeitfolgen von Krebserkrankungen

**DOI:** 10.1007/s00103-022-03506-1

**Published:** 2022-03-17

**Authors:** Joachim Weis

**Affiliations:** Stiftungsprofessur Selbsthilfeforschung, Comprehensive Cancer Center Freiburg, Medizinische Fakultät, Universitätsklinikum Freiburg, Hugstetter Str. 49, 79106 Freiburg, Deutschland

**Keywords:** Krebs, Psychische Befindlichkeit, Rezidivangst, Depression, Tumorassoziierte Fatigue, Neuropsychologische Defizite, Cancer, Psychosocial wellbeing, Fear of recurrence, Depression, Cancer-related fatigue, Neurocognitive dysfunctions

## Abstract

Die Inzidenz von Krebserkrankungen hat in den westlichen Industrienationen in den letzten Jahrzehnten stetig zugenommen. Die Anzahl der Neuerkrankungen liegt in Deutschland aktuellen Schätzungen zufolge bei ca. 500.000 pro Jahr. Aufgrund der verbesserten Früherkennung sowie der Fortschritte in den Behandlungsmöglichkeiten haben sich jedoch die Überlebenszeiten bei den meisten Tumorarten erhöht. In der Folge hat auch die Zahl der Langzeitüberlebenden (≥ 5 Jahre nach Diagnose oder Ende der Behandlung) zugenommen. Trotz der Erfolge der Tumortherapie können Langzeitüberlebende von verschiedenen körperlichen oder seelischen Problemen in der Folge der Erkrankung und/oder Therapie betroffen sein. Dieser Artikel gibt einen Überblick über die psychischen Folgeprobleme, insbesondere Angst, Depression, psychosoziale Aspekte der Lebensqualität, neuropsychologische Defizite sowie Erschöpfungszustände (Fatigue). In einem abschließenden Fazit werden Empfehlungen für psychosoziale Interventionen sowie für die Verbesserung der psychosozialen Versorgung von Langzeitüberlebenden gegeben.

## Abstract (en)

Incidence rates for cancer have increased in Western countries in recent decades, and the number of new cases in Germany is estimated at about 500,000 per year. Due to early detection of cancer and improved treatment strategies, survival time has improved for most cancer diagnoses. Therefore, the number of long-term cancer survivors (≥ 5 years after diagnosis) has also increased. Despite the advances in oncology, a substantial percentage of long-term survivors show reduced quality of life and suffering from various sequelae of cancer and its treatment. This review focuses on the most common psychological issues of long-term survivors such as anxiety and depression, psychosocial aspects of quality of life, neuropsychological deficits, and fatigue. Finally, recommendations are given for psychosocial interventions and improvements to the psychosocial care of long-term survivors.

## Einführung

In Deutschland wie in anderen westlichen Industrienationen sind über die letzten Jahre steigende Inzidenzzahlen für Krebserkrankungen festzustellen; nach neuesten Hochrechnungen des Robert Koch-Instituts (RKI) aus dem Jahr 2019 erkranken jährlich ca. 500.000 Menschen an Krebs. Dabei variieren die Verteilung und Häufigkeit abhängig vom Geschlecht [1]. Aufgrund einer verbesserten Früherkennung und verbesserter Behandlungsmöglichkeiten (Operation, Bestrahlung, Chemotherapie, personalisierte Therapiestrategien) sind die Überlebenszeiten bei den meisten Tumorarten in den letzten Jahren deutlich angestiegen.

Nach Angaben des RKI finden sich die günstigsten 5‑Jahres-Überlebensraten mit jeweils ca. 90 % bei Erkrankungen wie dem Lippenkrebs, dem malignen Melanom der Haut sowie bei Hodenkrebs und Prostatakrebs, während sich die ungünstigsten Raten unterhalb von 20 % bei Lungenkrebs, Speiseröhrenkrebs oder Krebs der Bauchspeicheldrüse zeigen [1]. Insgesamt haben sich die Überlebensraten von Krebspatientinnen und -patienten in Deutschland erheblich verbessert. Ende 2017 lebten in Deutschland insgesamt 4,65 Mio. Menschen mit bzw. nach einer Krebserkrankung (2,10 Mio. männlich und 2,55 Mio. weiblich). Etwa 2 Drittel aller Krebsüberlebenden gelten dabei als „Langzeitüberlebende“ (Krebsdiagnose liegt bereits ≥ 5 Jahre zurück; [2]).

Diese insgesamt positive Entwicklung in der Onkologie hat jedoch auch eine Kehrseite verschiedener behandlungsbedingter körperlicher und psychosozialer Folgeprobleme sowie Spätfolgen. Vor diesem Hintergrund ist die Thematik der Krebsüberlebenden und ihrer Belastung durch Langzeitfolgen der Therapie vor allem in den letzten Jahren zunehmend in den Fokus des wissenschaftlichen Interesses gerückt und wird in zahlreichen Studien beschrieben. Die wissenschaftliche Bedeutung wird ersichtlich durch die seit 2007 herausgegebene internationale Fachzeitschrift *Journal of Cancer Survivorship*^1^, die sich der gesamten Breite der Thematik widmet. Auf nationaler Ebene hat im Jahr 2018 das Bundesministerium für Gesundheit in der Steuerungsgruppe des Nationalen Krebsplans die Expertenarbeitsgruppe „Langzeitüberleben nach Krebs“ (AG LONKO) eingerichtet [2].

Die Diagnose einer Krebserkrankung stellt für die meisten Menschen ein stark belastendes Lebensereignis dar, das sich nicht nur in der Situation der Neuerkrankung und der darauffolgenden Behandlung auswirkt. Die Betroffenen können über viele Jahre oder sogar lebenslang von den Langzeitfolgen begleitet werden. Der Begriff „Überlebende“ (Survivors) wird in der Literatur sehr unterschiedlich verwendet. In einer Analyse anhand von verschiedenen Studien stellen Marzorati et al. [3] fest, dass eine einheitliche Definition derzeit nicht existiert und der Begriff „Überlebende“ in seiner weitesten Auslegung eine Zeitspanne von der Diagnosestellung bis hin zum Langzeitüberleben ohne Krankheitsanzeichen umfasst. In der Onkologie besteht jedoch weitestgehend die Übereinkunft, dass der Begriff „Langzeitüberlebende“ ehemals Krebserkrankte charakterisiert, die mindestens 5 Jahre nach Diagnosestellung noch am Leben sind.

In diesem Artikel werden die psychischen Spätfolgen von Krebserkrankungen und -therapien behandelt. Explizit ausgenommen sind hierbei Langzeitüberlebende, die sich in einer palliativen Situation befinden. Die Übergänge zwischen Nebenwirkungen und Spätfolgen sind fließend; in der Onkologie werden Nebenwirkungen, die länger als 90 Tage nach Ende der Therapie anhalten, als Spätfolgen definiert. Langzeitfolgen können als direkte oder indirekte Folgeprobleme der Tumorerkrankung und/oder -behandlung auftreten. Der vorliegende Beitrag befasst sich mit den psychischen Folgeproblemen bei Langzeitüberlebenden. Hierbei wird der aktuelle Kenntnisstand dargestellt und abschließend werden Empfehlungen zur Weiterentwicklung der psychosozialen Versorgung gegeben.

## Psychische Folgeprobleme bei Krebskranken

Viele Betroffene erleben durch eine Krebserkrankung einen Verlust der körperlichen Integrität und der Unversehrtheit, die auch Auswirkungen auf das seelische Erleben haben. Infolge der körperlichen Beeinträchtigungen durch die Krankheit und infolge der unter Umständen langwierigen Therapiemaßnahmen müssen nicht selten Alltagsaktivitäten aufgegeben werden, zum Teil vorübergehend, zum Teil auch auf Dauer. Oft ist der ganze bisherige Lebensentwurf infrage gestellt. Lebensziele müssen überdacht oder neu gefunden werden. Hieraus können Prozesse der Trauer über den Verlust, aber auch Auflehnung, Hader und Wut über das als ungerecht erlebte Schicksal resultieren. Als psychische Langzeitfolgen sind vor allem Angst und Depression, Einschränkungen der Lebensqualität, neuropsychologische Defizite sowie Müdigkeit und Erschöpfung (Fatigue) von Bedeutung.

### Angst und Depression

Trotz der eingangs genannten Verbesserung in den Behandlungsmethoden und den dadurch verbesserten Heilungsraten ist die Diagnose einer Krebserkrankung für viele Betroffene mit Sterben und Tod assoziiert und bedingt Gefühle der Angst oder Depression. Dies liegt im Wesentlichen darin begründet, dass auch nach einer erfolgreichen Behandlung der Verlauf häufig unsicher bleibt, da auch im günstigsten Fall immer ein Risiko für ein Wiederauftreten der Erkrankung bestehen bleibt. Daher können Krebskranke im Längsschnittverlauf die Bedrohung durch Sterben und den Tod nicht komplett verleugnen oder verdrängen, auch wenn sie nicht tagtäglich ans Sterben denken. Diese Bedrohung kann leicht aktualisiert werden, zum Beispiel wenn jemand von der Krebserkrankung eines anderen Menschen erzählt, wenn ein Nachuntersuchungstermin ansteht oder zum Jahrestag des Diagnosezeitpunkts. Als psychische Langzeitfolgen werden insbesondere Angst und Depression in der Literatur beschrieben, wobei es sich hier überwiegend um subklinische Ausprägungen handelt, die mithilfe von Fragebögen erfasst werden. Eine diagnostische Einordnung im Sinne der Klassifikation nach ICD (International Statistical Classification of Diseases) oder DSM (Diagnostic and Statistical Manual of Mental Disorders) erfolgt nur in wenigen Studien. Ebenso werden unterschiedliche Instrumente zur Erfassung eingesetzt, was teilweise die Vergleichbarkeit der Daten etwas erschwert.

Die Angst bei onkologischen Patientinnen und Patienten wird durch unterschiedliche Aspekte beeinflusst, wobei insbesondere Prädispositionen, frühere Erfahrungen mit Krebserkrankungen, die Krankheitsverarbeitung sowie die Intoleranz gegenüber Unsicherheit eine wichtige Rolle spielen [4]. Bei Langzeitüberlebenden spielt die Rezidiv- oder Progredienzangst eine besondere Rolle. Unter Rezidivangst wird die Angst vor dem erneuten Auftreten der Krebserkrankung, unter Progredienzangst die Angst vor dem Fortschreiten der Krebserkrankung verstanden. Da diese Formen der Angst einen realen Grund haben können, werden sie von neurotischen Ängsten oder sonstigen Angststörungen unterschieden.

In einem Review von k = 130 Studien war die Rezidivangst bei Langzeitüberlebenden eine der 10 häufigsten Belastungen und der am häufigsten genannte ungedeckte Versorgungsbedarf [5]. Die Ergebnisse zeigen, dass die vorhandene Rezidivangst über den gesamten Verlauf relativ stabil bleibt. Höhere Werte in der Rezidivangst sind assoziiert mit jüngerem Alter, Vorliegen von schweren körperlichen Symptomen, psychologischem Stresserleben und einer geringeren Lebensqualität. In der Folge zeigen sich Auswirkungen auf das Gesundheitsverhalten, psychologische Reaktionen und Funktionseinschränkungen. Aufgrund von heterogenen methodischen Ansätzen zur Erfassung der Rezidivangst lassen sich in diesem Review keine Angaben zur Prävalenz machen.

Koch et al. [6] analysieren auf der Basis von populationsbasierten Kohorten von Langzeitüberlebenden die Rezidivangst bei ehemaligen Brustkrebspatientinnen in Deutschland. Insgesamt konnten 2671 Frauen nach Brustkrebs einbezogen werden. Wenngleich die Mehrzahl der befragten Frauen eine geringe Rezidivangst (82 %) berichtet, litt ein substanzieller Prozentsatz von 11 % unter moderater Rezidivangst und 6 % unter hoher Rezidivangst. Auch hier zeigen sich wieder als Risikofaktoren ein jüngeres Lebensalter (Odds Ratio = 3,00, für Frauen < 55) sowie die subjektive Einschätzung, sich weiterhin als Tumorpatientin zu betrachten (Odds Ratio = 3,36). Ebenso waren höhere Werte der Rezidivangst mit höherer Depressivität sowie geringerer Lebensqualität assoziiert.

Zur Angstsymptomatik allgemein sowie zur Depression liegen einige Reviews sowie vereinzelt Metaanalysen für Langzeitüberlebende nach einer Krebserkrankung vor. Brandenbarg et al. [7] analysierten k = 20 Studien mit insgesamt 17.726 Patientinnen und Patienten mit Brust‑, Prostata- oder Darmkrebs. Die Depression wurde meist mit dem Fragebogen *Hospital Anxiety and Depression Scale* (HADS) gemessen, ansonsten finden sich Verfahren wie Brief Symptom Inventory (BSI), Becks Depressionsinventar (BDI) oder der Personal Health Questionnaire (PHQ). Die Angst wurde am häufigsten mit der HADS-Subskala (HADS-A) gemessen, weiterhin wurden der BSI (Subskala Angst) oder andere Verfahren eingesetzt. Auf Grundlage der gepoolten Daten wird für die Depression eine Prävalenz von 21,0 % (Varianz von 5,4–49,0 %), für die Angst 21 % (Varianz von 3,4–43,0 %) und für die allgemeine psychische Belastung 7 % (Varianz von 4,3–11,6 %) ermittelt.

Etwas niedrigere Angaben zu Angst und Depression finden sich in der Metaanalyse von Mitchell et al. [8], in der k = 144 Studien in das systematische Review einbezogen wurden und k = 43 in eine Metaanalyse eingeflossen sind. Die Prävalenz für die Depression wird mit 11,6 % (95 % KI 7,7–16,2), in der gepoolten Analyse 10,2 % (95 % KI 8,0–12,6) beziffert. In der gesunden Vergleichsstichprobe lagen die Werte bei einem relativen Risiko (RR) von 1,11. Die Prävalenz der Angst war mit 17,9 % (95 % KI 12,8–2,6) im Vergleich zu 13,9 % (95 % KI 9,8–18,5) einer gesunden Vergleichsstichprobe höher (RR = 1,27). Maass et al. [9] analysierten k = 17 Studien mit insgesamt *N* = 12.499 Frauen. In diesem Review werden jedoch auch Studien ab einem Jahr nach Diagnosestellung einbezogen, was die höheren Prävalenzwerte (Depression 9,4–66,1 %; Angst: 17,9–33,3 %) erklärt.

Neben Daten aus Metaanalysen und Reviews ergeben sich noch interessante Hinweise aus Verlaufsstudien. So fanden Oancea und Cheruvu [10] in einer Kohortenstudie zur Depression (gemessen mit PHQ-9 ≥ 10) bei erwachsenen Krebsüberlebenden (*N* = 2145 > 5 Jahre post Diagnose) eine Prävalenz von 10,1 %. Götze et al. [11] analysierten Angst und Depression in 2 Kohorten mit insgesamt *N* = 1002 Patientinnen und Patienten mit gemischten Krebsdiagnosen und verglichen sie mit einer Kontrollgruppe. Die erste Kohorte wurde 5 Jahre nach Krebsdiagnose, die zweite Kohorte 10 Jahre nach Krebsdiagnose untersucht. Die Depression wurde mit dem PHQ‑9, die Angst mit dem GAD‑7 erhoben. Für die Depression fand sich bei den Krebsüberlebenden eine Prävalenz von 12,5 % für moderate und 4,5 % für schwere Depressionen, wobei M (SD) bei Kohorte 1 = 5,29 (4,6) M (SD), bei Kohorte 2 = 4,85 (4,1) lag. Besonders hohe Werte hatten Brustkrebsüberlebende sowie jüngere Patientinnen und Patienten zwischen 23 und 60 Jahren. Bei Angst fand sich eine Prävalenz von 7,4 % für eine moderate und 1,6 % für eine schwere Ausprägung. Wiederum zeigten sich im Vergleich der Kohorten leichte Unterschiede zwischen Kohorte 1 = 4,00 (4,0) M (SD) und Kohorte 2 = 3,68 (3,8). Besonders hohe Angstwerte traten bei Hautkrebsüberlebenden und über alle Diagnosegruppen hinweg bei den jüngeren Patientinnen und Patienten (23- bis 60-Jährige) auf.

Es finden sich jedoch auch Hinweise, dass sich mit zunehmendem Abstand vom Ende der Behandlung das emotionale Befinden von Langzeitüberlebenden im Mittel verbessert. So fiel bei Brustkrebspatientinnen die Prävalenz einer depressiven Störung von 33 % zum Diagnosezeitpunkt auf 15 % im fünften Jahr nach Diagnose und entsprach damit dem Wert in der Allgemeinbevölkerung [12]. Die meisten depressiven Episoden lösten sich etwa 4 Monate nach der Diagnose auf, mit einer weiteren Verringerung nach dem zweiten Jahr. In einer anderen Studie wiesen nur noch 11 % der Betroffenen 4 Jahre nach Behandlungsabschluss in einem Fragebogen auffällige Werte zum emotionalen Befinden (HADS) auf [13].

Neben Folgeproblemen wird in der Literatur jedoch auch berichtet, dass nach schweren Lebenskrisen oder infolge von schweren Krankheiten positive Veränderungen etwa in Richtung einer verstärkten Sinnsuche möglich sind [14–16]. Unter dem Begriff des „posttraumatischen Wachstums“ [17] werden positive Anpassungs- und Veränderungsprozesse beschrieben. So untersuchten Liu et al. [18] in einer bevölkerungsbasierten Studie an 6952 Langzeitüberlebenden nach Brustkrebs, kolorektalem Krebs oder Prostatakrebs, inwieweit sie nach der Diagnose der Krebserkrankung positive Veränderungsprozesse sowie ein posttraumatisches Wachstum erleben. Die Ergebnisse zeigten, dass insgesamt 66,0 % der Befragten eine moderate bis hohe positive Veränderung in ihrem Leben berichteten und 20,5 % ein moderates bis hohes posttraumatisches Wachstum berichteten. Beide Aspekte waren von der Diagnose (Brustkrebs > kolorektales Karzinom > Prostatakrebs) und dem Geschlecht (Frauen > Männer) abhängig. Ebenso waren sie bei jüngeren Patientinnen und Patienten stärker ausgeprägt als bei älteren. Interessanterweise ergaben sich für das posttraumatische Wachstum keine Unterschiede zwischen Langzeitüberlebenden ohne Krankheitsanzeichen und denjenigen, die ein Rezidiv erlebt hatten. Ebenso zeigte sich, dass Brustkrebspatientinnen im Vergleich zu Patienten und Patientinnen mit kolorektalem Karzinom sowie Patienten mit Prostatakarzinom höhere Werte im Hinblick auf posttraumatisches Wachstum aufwiesen. In einer älteren Studie an Langzeitüberlebenden einer Brustkrebserkrankung im Vergleich zu einer Gruppe von Frauen, die ein anderes belastendes Lebensereignis erlitten hatten, gaben Brustkrebspatientinnen ein stärkeres posttraumatisches Wachstum an, obwohl sie mehr körperliche Folgewirkungen hatten, während sich in der psychischen Belastung keine Unterschiede zwischen beiden Gruppen nachweisen ließen [19].

Fasst man die Befundlage zusammen, so lässt sich festhalten, dass insbesondere die Rezidivangst ein Problem der Langzeitüberlebenden darstellt, wobei genaue Prävalenzangaben für diese Form der subsyndromalen Belastung schwer abzuschätzen sind. Insgesamt korreliert bei Langzeitüberlebenden Angst mit Depression, wobei die Angstwerte häufig höher liegen als die Depressionswerte. Die Prävalenzwerte für beide Bereiche schwanken zwischen 7 % und mehr als 20 %, hierbei finden sich zumeist höhere Werte für jüngere Patienteninnen und Patienten.

### Lebensqualität bei Langzeitüberlebenden

Die Lebensqualität ist ein geeigneter Indikator für die Identifizierung von Patientinnen und Patienten, die auch mehr als 5 Jahre nach der Diagnose noch unter verschiedenen körperlichen oder seelischen Einschränkungen leiden. Die Lebensqualität als multidimensionales Konstrukt umfasst neben seelischen und körperlichen Symptomen auch die verschiedenen Funktionsbereiche wie soziale, emotionale, physische oder kognitive Funktionen oder die Rollenfunktion [20]. Das Konzept ist daher sehr gut geeignet, die Probleme von Langzeitüberlebenden zu erfassen. In zahlreichen Studien konnte dargelegt werden, dass Krebsüberlebende, die kurativ behandelt wurden und tumorfrei sind, nicht einfach wieder „gesund“ sind, sondern an langfristigen Folgeproblemen leiden können.

Hierbei ist zunächst festzuhalten, dass die Lebensqualität langzeitüberlebender Krebskranker für die Mehrzahl der Befragten insgesamt als mit der Allgemeinbevölkerung vergleichbar bewertet wird, dennoch werden je nach Diagnosegruppe und Art der Behandlung unterschiedliche Probleme der Lebensqualität berichtet. Eine ältere Übersichtsarbeit analysierte 52 Studien zu 7 Diagnosegruppen (Brustkrebs, Eierstockkrebs, Gebärmutterhalskarzinom, Prostatakarzinom, kolorektales Karzinom, Kopf-und-Hals-Tumore, Morbus Hodgkin) im Hinblick auf die Lebensqualität von Langzeitüberlebenden [21]. Hierbei zeigten sich sehr unterschiedliche Ergebnisse im Vergleich der verschiedenen Diagnosegruppen. Bei Brustkrebs nahm die Lebensqualität im Verlauf der Zeit leicht zu, während sie bei Prostatakarzinompatienten abnahm. Probleme der Sexualität fanden sich insbesondere bei Frauen nach Brustkrebs, Zervix- oder Ovarialkarzinom sowie Hodgkin-Lymphomen. Menopausale Symptome wirkten sich negativ auf die psychologischen und sozialen Dimensionen der Lebensqualität aus. Über alle Diagnosegruppen waren Erschöpfungszustände (Fatigue) eines der häufigsten Langzeitprobleme, wobei insbesondere Brustkrebs‑, Ovarialkrebs- und Hodgkin-Lymphomüberlebende angaben, dass die Fatigue sich über die Zeit nicht verbesserte (siehe auch Abschnitt zu Fatigue). Ein Risikofaktor für eine Einschränkung der Lebensqualität war das jüngere Alter der Patientinnen.

Arndt et al. [22] untersuchten insgesamt 6952 Langzeitüberlebende in Deutschland 5–16 Jahre nach Diagnose einer Brustkrebs‑, Darmkrebs- oder Prostatakrebserkrankung im Rahmen einer epidemiologischen populationsbasierten Studie und verglichen sie mit einer Stichprobe von 1878 Menschen aus der Normalbevölkerung ohne Krebserkrankung. Die Lebensqualität wurde mit dem Fragebogen QLQ-C30 der European Organisation for Research and Treatment of Cancer (EORTC) erfasst und die Ergebnisse im Hinblick auf Alter, Geschlecht und Bildung kontrolliert. Insgesamt zeigt sich, dass die Lebensqualität bei Langzeitüberlebenden mit den Normwerten der gesunden Normalbevölkerung vergleichbar war, allerdings zeigten sich spezifische Defizite in der sozialen, emotionalen, kognitiven und körperlichen Funktion sowie der Rollenfunktion. Bei den Symptomen waren vorrangig Schlafstörungen, Fatigue sowie weitere körperliche Symptome häufiger und stärker ausgeprägt [22]. Besonders davon betroffen waren Krebsüberlebende jüngeren Lebensalters (< 50 Jahre). Analysen im Längsschnittverlauf zeigten nichtsignifikante Verschlechterungen bei sehr jungen und sehr alten Langzeitüberlebenden.

Fasst man die Befundlage zusammen, so zeigt sich für die Mehrzahl der Langzeitkrebsüberlebenden eine der Normalbevölkerung vergleichbare Lebensqualität. Allerdings zeigen die Daten auch, dass Subgruppen über lange Zeit deutliche Einschränkungen in der Lebensqualität aufweisen. Dass Krebskranke ihre Lebensqualität teilweise sogar besser bewerten als Gesunde, ist ein auf den ersten Blick paradox anmutendes Phänomen, jedoch in der Lebensqualitätsforschung bekannt [23]. Dieses Paradox kann als Folge einer Verschiebung des Bewertungsmaßstabs (Response Shift) verstanden werden, an dem die Lebenszufriedenheit beurteilt wird. Der Response Shift umfasst hier sowohl ein „Herabsetzen“ des eigenen Anspruchsniveaus als auch eine neue Einordnung der unterschiedlichen Lebensbereiche hinsichtlich ihrer Bedeutung für das Zufriedensein.

Nach Schwartz et al. [24] lassen sich 3 verschiedene Arten von Response Shift beschreiben: Rekalibrierung, Repriorisierung und Neukonzeptualisierung. Bei der *Rekalibrierung* wird die Bewertungsskala, das heißt der Maßstab verändert, mit dem die eigene Lebensqualität bewertet wird: Was zuvor noch unerträglich erschien, wird nun als bewältigbar wahrgenommen. Bei der *Repriorisierung* wird die Bedeutung einzelner Dimensionen der Lebensqualität verändert: Die körperliche Leistungsfähigkeit verliert möglicherweise an Bedeutung, enge persönliche Beziehungen werden wichtiger. Mit der *Neukonzeptualisierung* bewerten die Betroffenen die verschiedenen Dimensionen der Lebensqualität neu, sodass Bereiche, die vorher genuine Bestandteile der Lebensqualität waren, wie zum Beispiel der Beruf, in ihrer Bedeutung geringer werden, während neue, wie zum Beispiel kulturelle Interessen oder soziale Beziehungen an Bedeutung gewinnen. Insofern ist dieser Response Shift auch ein Ausdruck eines positiven Anpassungsprozesses an eine veränderte Lebenssituation und einer Neubewertung im Sinne des posttraumatischen Wachstums wie im Abschnitt oben ausgeführt. Insofern können Prozesse der Neubewertung eine günstige Wirkung auf die Lebensqualität haben, ohne dass es sich dabei um bloße „Messfehler“ handelt.

### Neuropsychologische Folgeprobleme

In den letzten 2 Jahrzehnten wurde den Therapiefolgestörungen in Bezug auf kognitive Funktionen wie Aufmerksamkeit, Gedächtnis- und exekutive Leistungen bei onkologischen Patientinnen und Patienten zunehmend Beachtung geschenkt. Die Neurotoxizität vieler onkologischer Therapien, speziell auch der spezifischen chemotherapeutischen Substanzen war schon lange Zeit bekannt, doch fokussierte die klinische Onkologie über viele Jahre weitgehend nur die neurologischen Aspekte, wie zum Beispiel die periphere Polyneuropathie (Missempfindungen, Taubheitsgefühle oder auch schmerzhafte Zustände in den peripheren Gliedmaßen vor allem an den Händen und Füßen), oder auf massive Beeinträchtigungen des zentralen Nervensystems wie Leukenzephalopathien oder die Hirnnerven betreffende Störungen (zum Beispiel Schädigungen der Hörnerven infolge verschiedener Substanzen).

Wissenschaftliche Studien zur Erfassung neurokognitiver Störungen bei Krebserkrankten wurden überwiegend mit Brustkrebspatientinnen durchgeführt [25]. In einer Übersicht zum aktuellen Kenntnisstand [25], insbesondere zu Studien an Brustkrebspatientinnen, lässt sich zusammenfassen, dass sich die Einschränkungen vorrangig auf die Gedächtnisleistung, die kognitive Verarbeitungsgeschwindigkeit, die Aufmerksamkeit und auf exekutive Funktionen beziehen. Studien zeigen, dass auch die Hormontherapie oder die neuen zielgerichteten Therapiestrategien kognitive Probleme nach sich ziehen können [26, 27]. Hinsichtlich der prädisponierenden Faktoren der neurokognitiven Funktionen werden das Alter, genetische Polymorphismen bestimmter biologischer Marker und Neurotrophine diskutiert und mit einem erhöhten Risiko in Zusammenhang gebracht [28]. Schlechte Leistungen in neuropsychologischen Tests sind in einigen Studien mit einer Volumenreduktion der grauen Hirnmasse sowie geringerer Hirnaktivität nach der Chemotherapie assoziiert. Aus Tierversuchen ergeben sich Hinweise, dass auch eine veränderte Neurogenese, eine mitochondriale Dysfunktion oder Zytokinreaktionen des Gehirns dafür verantwortlich sein könnten, wobei definitive Erklärungen für Ursache-Wirkungs-Zusammenhänge bislang noch ausstehen [28].

Neuropsychologische Defizite als mögliche Langzeitfolgen der onkologischen Therapie sind in den letzten beiden Jahrzehnten systematisch untersucht worden [29]. Hierbei werden die neuropsychologischen Folgeprobleme einerseits über die subjektiv erlebten Einschränkungen mittels Fragebögen erfasst, andererseits über neuropsychologische Tests. In der Regel sind die Prävalenzzahlen auf der Basis von subjektiv berichteten Beschwerden höher als die auf der Basis neuropsychologischer Tests ermittelten Auffälligkeiten. So fanden sich subjektive berichtete kognitive Beschwerden bei Brustkrebspatientinnen nach Chemotherapie bei mehr als 50 % der Frauen, während nur 15–25 % objektive kognitive Defizite aufwiesen [25]. Als mögliche Erklärung wird davon ausgegangen, dass die subjektiv wahrgenommenen Leistungseinschränkungen von Angst und Depression sowie dem psychologischen Belastungserleben beeinflusst werden [30–32].

Da Einschränkungen, die bereits vor der Erkrankung und Therapie bestanden, zumeist nicht bekannt sind und die kognitiven Folgeprobleme bisher nur in wenigen prospektiven Studien untersucht wurden, ist der auf die onkologische Therapie zurückzuführende Anteil an diesen Folgestörungen nicht eindeutig abzuschätzen. Erfahrungen aus zahlreichen prospektiven Studien deuten auf eine multifaktorielle Genese hin, in der neben möglichen direkten neurotoxischen Effekten auch psychologische Faktoren eine Rolle spielen [33]. In einer prospektiven Längsschnittstudie wurden 136 Überlebende einer Brustkrebserkrankung mehr als 5 Jahre nach Ende der Behandlung sowohl mit neuropsychologischen Testbatterien als auch mit Fragebögen zur Erfassung der subjektiven Beschwerden untersucht [34]. Insgesamt 15 % der Stichprobe wiesen in den Tests Einschränkungen in mehr als 2 Funktionsbereichen auf und 32 % in einem Funktionsbereich. Die subjektive Wahrnehmung der kognitiven Funktion war im Vergleich zu Normdaten signifikant schlechter. Subjektive und objektive Maße der kognitiven Funktion korrelierten stark (*p* = 0,006). Deutlich höhere Werte fanden sich für Langzeitüberlebende nach erfolgreicher hämatologischer Stammzelltransplantation. Trotz deutlicher Verbesserungen in nahezu allen kognitiven Funktionsbereichen im Vergleich zum Zeitpunkt 80 Tage nach Stammzelltransplantation zeigten in einer prospektiven Längsschnittstudie 5 Jahre danach in der Summe aller Funktionsbereiche 41,5 % der Überlebenden milde oder stärker ausgeprägte Defizite im Vergleich zu 19,7 % einer Kontrollgruppe mit populationsbasierten Normwerten [35].

Insgesamt wird die Prävalenz neuropsychologischer Defizite als Langzeitfolge mit Werten zwischen 15 % bis über 40 % in der Literatur angegeben. Aufgrund der engen Zusammenhänge mit dem psychischen Belastungserleben und der wenigen vorliegenden Daten zur Testdiagnostik im Längsschnittverlauf ist das Problem neurokognitiver Störungen als Langzeitfolge schwer zu bewerten. Aufgrund der bestehenden Datenlage kann davon ausgegangen werden, dass ein Prozentsatz von ca. 15 % in Abhängigkeit von der Grunderkrankung sowie der Art der Behandlung unter objektivierbaren Einschränkungen leidet.

### Tumorassoziierte Fatigue

Eine anhaltende Erschöpfung im Zusammenhang mit einer Krebserkrankung wird als tumorassoziierte Fatigue bezeichnet und gilt als eines der häufigsten Folgeprobleme einer Tumorerkrankung beziehungsweise deren Behandlung. Die Fatigue wird in den meisten Definitionen als ein belastendes Gefühl atypischer Müdigkeit und Schwäche auf körperlicher, emotionaler und kognitiver Ebene beschrieben, die sich durch Ausruhen oder Schlaf nicht wesentlich verbessert [36].

Auf der körperlichen Ebene zeigt sich die Fatigue beispielsweise in einer reduzierten körperlichen Leistungsfähigkeit. Antriebs- und Interessenlosigkeit sind die häufigsten Symptome auf der emotionalen Ebene, während sich auf der kognitiven Ebene vor allem Konzentrationsschwierigkeiten und Probleme des Kurzzeitgedächtnisses zeigen. Fatigue kann zu jedem Zeitpunkt der Tumorerkrankung auftreten und führt zu starken Einschränkungen der Leistungsfähigkeit und Lebensqualität der Betroffenen [37]. Angaben zu den Prävalenzraten schwanken zwischen 59 % und mehr als 90 %. Von der tumorassoziierten Erschöpfung während oder unmittelbar nach Ende der Tumortherapie sind fast alle Patientinnen und Patienten betroffen, als Langzeitfolge tritt sie jedoch deutlich seltener auf. Für Fatigue als Langzeitfolge werden Prävalenzen zwischen 20 % und 50 % berichtet.

In einer Studie von Mehnert et al. [38] waren 5 Jahre nach Beendigung der Mammakarzinomtherapie im Durchschnitt noch ca. 50 % der Patientinnen betroffen. Diese Ergebnisse sind mit Befunden aus internationalen Studien vergleichbar, die zeigten, dass von Fatigue betroffene Langzeitüberlebende unter einer deutlich verminderten Lebensqualität litten. Als Langzeitfolge werden Prävalenzwerte zwischen 25 % und 35 % angegeben [36, 39, 40].

Die tumorassoziierte Fatigue kann als Langzeitfolge der Erkrankung und Behandlung auftreten, wobei sowohl das Fortbestehen der Problematik über mehr als 2 Jahre als auch das neue Auftreten einige Jahre nach Therapieende zusammengefasst werden. Insbesondere nach hämatologischer Stammzelltransplantation ist die Fatigue eine häufige und für eine Teilgruppe der Betroffenen lang andauernde Folgeproblematik [41, 42]. Langzeitüberlebende nach einer Morbus-Hodgkin-Erkrankung zeigen über viele Jahre erhöhte Fatiguewerte [43]. In einer registerbasierten Langzeitstudie fanden Oerlemans et al. [44], dass nach einem Zeitraum von 10 Jahren 6 von 10 Lymphomerkrankte unter verstärkter Fatigue litten. Einflussfaktoren waren das Stadium der Erkrankung bei Diagnosestellung (Stadium IV) sowie das Vorhandensein von physischen und psychischen Komorbiditäten. Vistad et al. [45] fanden, dass bei einer Gruppe von Patientinnen mit Zervixkarzinom 5 Jahre nach Behandlung (Operation und Bestrahlung) noch 30 % unter Fatigue litten. Verglichen mit der Allgemeinbevölkerung hatten diese Patientinnen eine signifikant schlechtere Lebensqualität, stärkere körperliche Beeinträchtigungen sowie höhere Werte für Angst und Depression. In einem multivariaten Regressionsmodell war die Depression als einzige Variable mit der Fatigue bei den Langzeitüberlebenden assoziiert.

Trotz unklarer Genese und des Fehlens eines umfassenden Erklärungsmodells werden die Zusammenhänge zwischen der Tumorerkrankung beziehungsweise deren Behandlung und der Fatigue als Spätfolge kontrovers diskutiert. Aufgrund der unklaren Ursache-Wirkungs-Zusammenhänge wird die Langzeitfatigue auch im Kontext einer maladaptiven Krankheitsverarbeitung interpretiert. Nach heutigem Verständnis ist die Fatigue als multidimensionales Syndrom zu verstehen, das somatische und psychologische Faktoren sowie Verhaltensaspekte umfasst. Die Fatigue als Langzeitfolge hat auch Auswirkungen auf die Gesundheitsversorgung sowie die im Gesundheitssystem entstehenden direkten oder indirekten Kosten. Patientinnen und Patienten, die unter Fatigue leiden, nehmen häufiger ärztliche und andere Gesundheitsdienste in Anspruch und bei ihnen finden sich höhere Raten an Arbeitsausfällen, Einbußen in der Arbeitsfähigkeit sowie vorzeitige Berentung [46].

In Tab. 1 werden die geschätzten Prävalenzangaben der psychischen Langzeitfolgen nach einer Krebserkrankung als Übersicht dargestellt.

**Table 1:** 

Symptome	Geschätzte Prävalenz^a^ (in %)
Angst	3–43
Depression	5–66
Allgemeine psychische Belastung	4–12
Neuropsychologische Defizite	15–40
Fatigue	20–50

^a^Prozentangaben auf der Basis von Daten aus Reviews und Metaanalysen

## Assessmentverfahren zur Erfassung psychischer Langzeitfolgen

Psychische Langzeitfolgen können über unterschiedliche Instrumente erfasst werden. Die oben beschriebenen Probleme der Angst, Depression und Fatigue werden über entsprechende Instrumente wie HADS, PHQ‑9, GAD‑7 und das Distress-Thermometer ermittelt und verfolgen einen epidemiologischen Ansatz der Einordnung in Schwellenwerte oder des Vergleichs mit Normwerten. Für diese Verfahren liegen deutschsprachige standardisierte und validierte Instrumente vor [47]. Darüber hinaus besteht die Möglichkeit, die psychischen Problemlagen von Langzeitüberlebenden strukturiert über die Bedürfnisse und Anliegen von Betroffenen zu erfassen, die vor allem im Hinblick auf den Versorgungsbedarf und die Entwicklung von Interventionen von Bedeutung sind [48–50].

Die überwiegend in den USA entwickelten Verfahren zur Bedarfserfassung umfassen auch die hier behandelten Symptomkomplexe und liegen teilweise auch als Kurzform vor. Leider stehen diese Verfahren bislang nicht in einer deutschsprachigen Validierung zur Verfügung.

Ein weiterer Ansatz ist die Erfassung der Lebensqualität. Hierbei wurde deutlich, dass die gesundheitsbezogene Lebensqualität von Langzeitüberlebenden ohne Krankheitsanzeichen nur begrenzt mit den vorliegenden Instrumenten erfasst werden kann, da die krebsspezifischen Verfahren primär für die Messung von Symptomen entwickelt wurden, die durch die akute Erkrankung und Behandlung bedingt sind [20]. Daher wurden einige Verfahren entwickelt, die die Lebensqualität von Langzeitüberlebenden abbilden. Einen Literaturüberblick geben Muzzatti und Annunziata [51]. In der Regel handelt es sich dabei jedoch um umfangreiche multidimensionale Instrumente, die in Teilen auch die hier behandelten psychischen Folgeprobleme erfassen [52–54]. Ein von der EORTC entwickeltes Instrument zur Erfassung der Lebensqualität bei Langzeitüberlebenden nach einer Krebserkrankung liegt in der ersten Version in Form eines übergreifenden Kernfragebogens vor [55].

## Fazit

Wie in der vorliegenden Übersicht zusammenfassend dargestellt wurde, nimmt die Zahl der Langzeitüberlebenden nach einer Tumorerkrankung, bedingt durch die verbesserte Früherkennung und Behandlung, in den letzten Jahren stetig zu. Zugleich wird deutlich, dass Langzeitprobleme in verschiedenen psychologischen Funktionsbereichen, in der Lebensqualität, in neurokognitiven Einschränkungen sowie in Form einer Fatigue auftreten können. Auch wenn gezeigt werden konnte, dass sich die Lebensqualität für die Mehrzahl der Patientinnen und Patienten nach Beendigung der Therapie im Zeitverlauf deutlich verbessert, weist ein substanzieller Teil verschiedene psychosoziale Folgeprobleme auf. Insofern ist die Identifikation der Häufigkeit und Ausprägung psychischer Langzeitfolgen eine wesentliche Voraussetzung dafür, den möglichen Bedarf für auf diese Zielgruppen ausgerichtete Programme wie Psychoedukation, Bewegungs- oder Psychotherapie eruieren zu können. Dadurch können wichtige Daten über die Bedürfnisse der ehemaligen Patientinnen und Patienten und erste Schätzungen für den Bedarf an notwendigen Interventionen oder Programmen ermittelt werden. Für die in diesem Beitrag behandelten psychologischen Problembereiche verfügen wir mit leichten Einschränkungen für den Bereich der Bedarfsermittlung bereits über validierte Screeningverfahren zur subjektiven Erfassung von Angst, Depression, Lebensqualität, neuropsychologischen Einschränkungen und Fatigue, die einen ersten Schritt zur Identifizierung von unterstützungsbedürftigen Langzeitüberlebenden darstellen können. Vor dem Hintergrund der aufgezeigten Problembereiche ist es jedoch eine große Herausforderung, psychosoziale Interventionen oder Konzepte einer Spätrehabilitation für Langzeitüberlebende zu entwickeln und Beratungs- und Schulungsangebote zu etablieren. Aufgrund der Komplexität der dargestellten Probleme wird es nicht immer möglich sein, eine vollständige Wiederherstellung der Gesundheit zu erreichen, jedoch sind eine Symptomlinderung und das Erlernen von Methoden zum besseren Umgang mit den Einschränkungen ebenfalls relevante Ziele entsprechender Interventionsprogramme.

## Footnotes

1 https://www.springer.com/journal/11764.
